# Optimal waiting period for frozen embryo transfer after hysteroscopic polypectomy: A propensity score matching analysis

**DOI:** 10.3389/fendo.2022.986809

**Published:** 2022-09-30

**Authors:** Bijun Wang, Nan Meng, Wenjuan Zhang, Pingping Kong, Zhaozhao Liu, Wenxia Liu, Huaqing Sun, Wen Zhang, Chenchen Ren, Yichun Guan

**Affiliations:** ^1^ Department of Obstetrics and Gynecology, The Reproductive Medicine Center, The Third Affiliated Hospital of Zhengzhou University, Zhengzhou, China; ^2^ Gynecology and Obstetrics, The Third Affiliated Hospital of Zhengzhou University, Zhengzhou Key Laboratory of Cervical Disease, National Clinical Research Center for Obstetrics and Gynecology, Henan Branch, Zhengzhou, China

**Keywords:** endometrial polyps, hsteroscopy, polypectomy, frozen embryo transfer, timing

## Abstract

**Objective:**

To evaluate the optimal waiting period for frozen-thawed embryo transfer (FET) after hysteroscopic polypectomy (HSC-P).

**Design:**

Retrospective cohort.

**Setting:**

University-affiliated hospital.

**Patient(s):**

All patients included in this research underwent hysteroscopy before the first FET cycle after whole embryo freezing. A total of 206 patients had undergone HSC-P, and 3681 patients without endometrial polyps were defined as the controls.

**Intervention(s):**

HSC-P.

**Main outcome measure(s):**

The HSC-P group was divided into three subgroups based on the time interval between HSC-P and the start of an FET cycle. Subgroup 1 consisted of patients who underwent FET after their next menses, subgroup 2 after two menstrual cycles, and subgroup 3 after three or more menstrual cycles. Demographics, baseline *in vitro* fertilization (IVF) characteristics, and pregnancy outcomes, especially perinatal outcomes after FET were compared among the groups.

**Results:**

There were 137 patients in subgroup 1, 40 in subgroup 2, and 29 in subgroup 3. There were no differences in the baseline characteristics of the three groups. IVF-related data and FET-related data, such as endometrial thickness and ET no. Of embryoes, were similar among the three subgroups. The three subgroups showed no significant differences in implantation rate, biochemical pregnancy rate, abortion rate, clinical pregnancy rate or live birth rate. Besides, There was no significant difference in perinatal outcomes including very preterm delivery, preterm delivery, low birth weight, macrosomia, small for gestational age, large for gestational age, birth weight(g), birth-height(cm)and Apgar Scores.

**Conclusion(s):**

Compared with FET after their next menses, FET after two or more menstrual cycles after HSC-P does not necessarily produce superior outcomes.

## Introduction

Endometrial polyps are a common gynaecological disease that might result in the reappearance of abnormal uterine bleeding or infertility (1). Three-dimensional ultrasound, saline infusion sonography, hysterosalpingography and hysteroscopy are all methods used to diagnose endometrial polyps; notably, saline infusion sonography and hysteroscopy have high sensitivity and specificity and are the preferred diagnostic methods (2). However, hysteroscopy remains the gold standard for diagnosis and treatment (3, 4). Endometrial polyps are growing tumours and thus produce significant plasma glycodelin levels, which may impair fertilization and implantation (5). Due to the possible effect of endometrial polyps on fertility, their removal prior to any subfertility treatment is widely practised.

Multiple clinical studies have shown that surgical removal of endometrial polyps in infertile patients prior to intrauterine insemination (IUI), *in vitro* fertilization (IVF) or intracytoplasmic sperm injection (ICSI) may improve reproductive outcomes (6–9). For instance, scholars have reported that a freeze-all strategy followed by hysteroscopic polypectomy (HSC-P) and vitrified-warmed embryo transfer (ET) is a viable option (10). However, the optimal interval between HSC-P and ET has not been conclusively determined. This may require consideration of the timing of endometrial repair and recurrence of endometrial polyps. The duration of endometrial wound healing is different after various surgeries, and most patients achieve a fully healed endometrium 1 month after HSC-P (11). Other studies have reported that a high number of endometrial polypectomy treatments, endometriosis, and previous polypectomy history are independent risk factors for recurrence after HSC-P, which becomes particularly pronounced after 1 year (12, 13). These data, however, do not inform the optimal timing of assisted reproductive technology (ART) transplantation.

Cohort studies have been performed to compare different intervals between fresh embryos transfer and HSC-P and have indicated that patients can undergo ovarian stimulation after their next menses without affecting IVF-ET outcomes (14, 15); in contrast, there are limited data regarding the optimal waiting period for frozen ET after HSC-P. Thus, we investigated whether the time interval between HSC-P and the start of frozen ET cycles affects reproductive outcomes.

## Materials and methods

### Patients

This retrospective, single-centre cohort study included ART treatment cycles carried out at the Reproduction Medicine Center of the Third Affiliated Hospital of Zhengzhou University between January 2015 and December 2020. The study was approved by the institutional review board of the Third Affiliated Hospital of Zhengzhou University.

None of the patients included in this research underwent hysteroscopy before the first IVF cycle, and all the patients underwent hysteroscopy before the first frozen-thawed embryo transfer (FET) cycle after whole embryo freezing. The exclusion criteria included the following: 1) oocyte donation, embryos from frozen-thawed oocytes, embryos cryopreserved for reasons related to malignancies, and cycles with preimplantation genetic testing; 2) known uterine anomalies, including endometritis, uterine malformation, and submucosal fibroid; 3) the presence of hydrosalpinx not corrected surgically prior to FET; and 4) uncontrolled endocrine or immune disorders or other systemic diseases, including hypertension, diabetes, thyroid disease, hyperprolactinemia, antiphospholipid syndrome, and systemic lupus erythematosus. Each patient signed an informed consent form prior to the initiation of IVF/ICSI-ET treatment allowing their clinical data to be obtained and analysed.

Endometrial polyps were diagnosed with the use of the criteria described by Perez-Medina et al. (6). The study group composed of patients who had received treatment with HSC-P after the first IVF cycle with whole embryo freezing but prior to FET. The control group was defined as patients with normal hysteroscopic results after the first IVF cycle with whole embryo freezing but prior to FET. Propensity score matching (PSM) was used to match the participants 1:1 based on age, body mass index (BMI), anti-Müllerian hormone (AMH) level, number of embryos transferred and type of embryos transferred (cleavage-stage embryos or blastocysts). All of the HSC-P treatments were performed in the operating room by the same physician (Huaqing Sun) in the follicular phase of the menstrual cycle. All endometrial polyps were confirmed histologically. Patients with any surgical intervention to the endometrium prior to FET that could cause endometrial damage were excluded.

**Figure 1:**
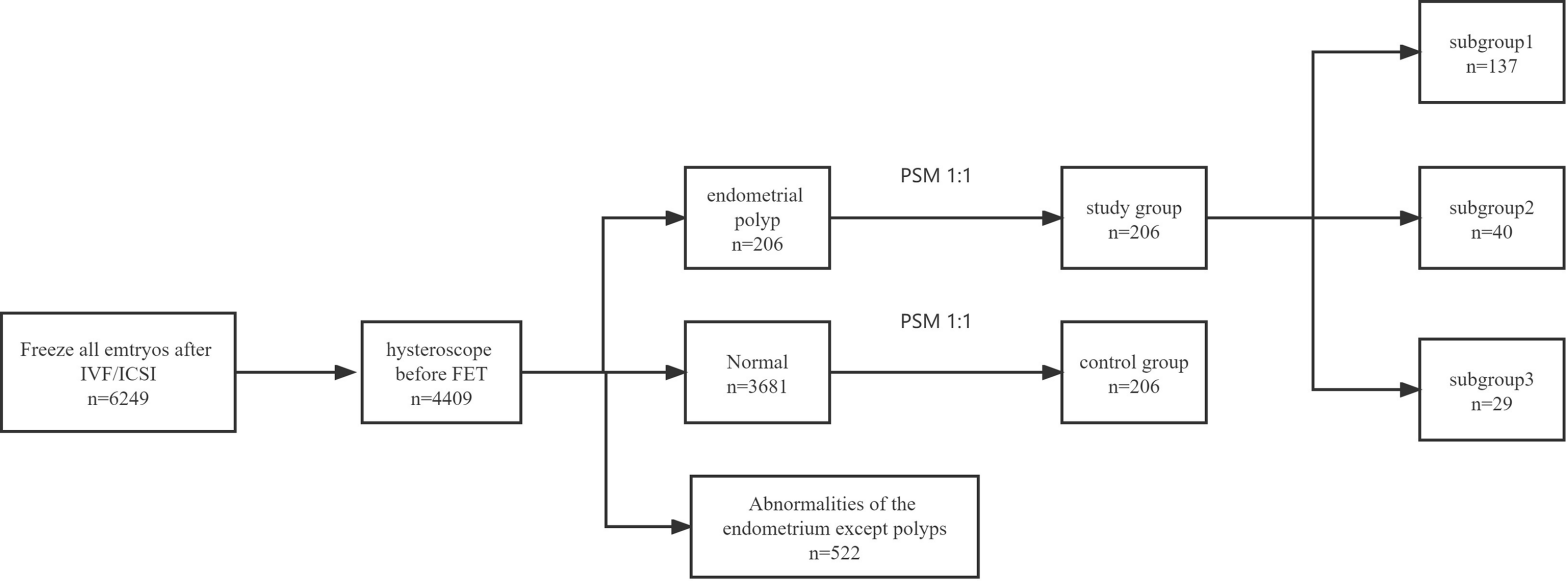
Flowchart of the included population.

By study design, the patients in the study group were assigned to three subgroups based on the time interval between HSC-P and the start of an FET cycle: subgroup 1 consisted of patients who underwent FET after their next menses, subgroup 2 after two menstrual cycles, and subgroup 3 after three or more than three menstrual cycles (**Figure 1**).

### Clinical and laboratory protocols

The women underwent standard controlled ovarian hyperstimulation (COH) with a downregulation protocol using a GnRH agonist or an antagonist protocol according to the patients’ conditions. For women with poor ovarian response, mild ovarian stimulation was offered, as described previously (16, 17). The physician adjusted the starting dose according to the patient’s age, BMI and ovarian reserve. Ovarian follicle development was monitored based on serum estradiol and transvaginal ultrasonographic measurements. If at least three follicles were > 18 mm in diameter, human chorionic gonadotropin (Ovidrel, Merck Serono, Germany) or a 0.2 mg dose of Triptorelin (Decapeptyl Daily, Ferring, Switzerland) was administered. Transvaginal oocyte retrieval was performed 36 h later. Embryos that were not suitable for cryopreservation on day 3 were cultured until days 5 or 6 and vitrified if they reached the blastocyst stage. FET was performed after preparation *via* hormone replacement therapy (HRT) or during a natural cycle (NC) (18). Vaginal or oral progesterone (Crinone, Merck Serono, Switzerland) was provided for luteal support.

### Outcome variables

The implantation rate was defined as the mean number of gestational sacs observed on transvaginal ultrasonography divided by the number of embryos transferred for each patient. The clinical pregnancy rate was defined as the number of intrauterine gestations with foetal cardiac activity per IVF-ET cycle. A biochemical pregnancy was defined as a positive hCG level without a gestational sac. Any pregnancy loss after visualization of intrauterine gestation was considered to be a spontaneous miscarriage, and any birth after 24 weeks of gestation was considered to be a live birth.

### Endometrial preparation for FET

Endometrial preparation for FET was achieved by NC or HRT programs. Ovulation in the NC program was determined by monitoring follicular development with transvaginal ultrasonography and hormone levels. The patients receiving HRT-FET cycles were treated with daily oral estradiol valerate tablets (Progynova, Bayer, Germany) starting on the second day of menstruation. When the endometrial thickness reached 7 mm or thicker, oral dydrogesterone (2 times daily, 10 mg once) (Abbott Co. USA) and intravaginal administration of 90 mg of a progesterone sustained-release vaginal gel (Merck Co. Germany) were given daily. One or two thawed embryos were transferred with ultrasound guidance on the third day (cleavage-stage embryo) or the fifth day (blastocyst) after ovulation or progesterone administration. All the patients received luteal support with progesterone after ET. If transvaginal ultrasound showed a gestational sac and embryonic heartbeat 1 month after ET, luteal support was continued until 2 months of gestational age.

### Statistical methods

Statistical analysis was performed using SPSS software (version 17.0 for Windows; SPSS Inc., Chicago, IL, USA). Normally distributed continuous variables are presented as the mean value ± standard deviation (SD). Nonnormally distributed continuous data are presented as the median and range. Categorical data are described by the number of cases, including numerator/denominator and percentages. A value of p<0.05 was considered significant. Continuous variables were calculated *via* dependent-sample t tests or Mann–Whitney U tests as appropriate. Categorical variables were analysed *via* the chi-square test or Fisher’s exact test as appropriate. PSM was utilized for sampling at up to 1:1 nearest-neighbour matching with calliper (0.05) to balance the baseline and improve the comparability between groups. The PSM allowed each patient who underwent HSC-P to be matched to patients without endometrial polyps after hysteroscopy with similar characteristics, which included age, BMI, ET no. Of embryos and ET levels of embryos (embryos or blastocyst). By using a previous study comparing the clinical pregnancy rate between women with FET after HSC-P and IVF/ICSI-ET (63% versus 41%) without polyps as a reference (10), a total of 206 participants (after PSM) would provide 95% power, assuming a standard deviation of 2 and an alpha of 0.05.

## Results

The clinical characteristics of the control group and the study group before and after PSM are shown in **Table 1**. There were no differences between the two groups in terms of age, BMI, gravidity, or peak estradiol level during the IVF/ICSI cycle. The reasons for whole embryo freezing in IVF cycles were as follows: patients in group 1 adopted the freeze-all embryos strategy due to the following conditions: 28.6% had a high risk of ovarian hyperstimulation syndrome (OHSS), 23.86% had premature progesterone elevation, 16.5% had abnormalities in the endometrium by ultrasound or inadequate endometrial thickness, 27.2% because of the protocol, and 3.9% under patient request. The patients in group 2 (after PSM) underwent the freeze-all embryos strategy and then FET for the following indications: 14.6% had a high risk of OHSS, 7.3% had premature progesterone elevation, 50% had abnormalities in the endometrium by ultrasound, 23.8% because of the protocol, and 4.3% under patient request.

**Table 1:** Baseline characteristics of all patients.

	Before PSM			After PSM		
	Group1: Controls (n = 3681)	Group2: Polypectomy (n = 206)	*p*	Group1: Controls (n = 206)	Group2: Polypectomy (n = 206)	*p*
Age (year)	31.53 ± 5.38	33.32 ± 5.39	<0.001	33.31 ± 5.63	33.32 ± 5.39	0.979
BMI (kg/m^2^)	23.72 ± 3.33	24.27 ± 3.61	0.034	24.12 ± 3.67	24.27 ± 3.61	0.691
Basal FSH (IU/L)	7.54 ± 2.94	7.10 ± 3.19	0.323	7.16 ± 3.35	7.10 ± 3.19	0.861
Basal LH(IU/L)	9.30 ± 17.42	7.92 ± 16.35	0.245	7.38 ± 12.01	7.92 ± 16.35	0.705
AMH (pmol/L)	20.07 ± 22.89	12.98 ± 16.23	<0.001	14.24 ± 18.04	12.98 ± 16.23	0.476
Duration of infertility (years)	3.39 ± 2.32	3.42 ± 2.55	0.781	3.87 ± 2.97	3.42 ± 2.55	0.875
Gravidity	1.01 ± 1.332	0.99 ± 1.39	0.843	1.24 ± 1.445	0.99 ± 1.39	0.072
Total dosage of Gn used (IU)	2458.75 ± 1002.00	2842.23 ± 1076.23	<0.001	2705.45 ± 1008.89	2842.23 ± 1076.23	0.184
Duration of Gn used (d)	12.13 ± 2.90	11.94 ± 2.94	<0.371	12.16 ± 2.97	11.94 ± 2.94	0.465
**OPU cycle**						
Peak E2(nmol/l)	18483.70 ± 6772.56	15895.43 ± 8217.54	<0.001	15807.52 ± 6035.77	15895.43 ± 8217.54	0.902
Number of oocytes Retrieved	16.20 ± 8.99	10.99 ± 6.55	<0.001	13.91 ± 8.62	10.99 ± 6.55	<0.001
Number of good-quality embryos	4.90 ± 4.43	3.26 ± 2.88	<0.001	3.12 ± 4.13	3.26 ± 2.88	0.112
Number of blastocysts	3.30 ± 2.66	2.81 ± 2.30	<0.001	.77 ± 2.95	1.81 ± 2.30	0.243
**Endometrial preparation**			0.593			0.795
Natural cycle	49.93% (1838/3681)	45.63% (94/206)		46.64% (96/206)	45.63% (94/206)	
Artificial cycle	50.06% (1843/3681)	54.37% (112/206)		53.36% (110/206)	54.37% (112/206)	
Endometrial thickness	9.45 ± 1.65	10.41 ± 1.96	0.529	9.66 ± 1.75	9.41 ± 1.96	0.634
ET no. Of embryoes	1.45 ± 0.49	1.54 ± 0.49	0.012	1.57 ± 0.49	1.54 ± 0.49	0.621
**Development stage of the embryo**						
D3	56.36% (3017/5353)	71.70%(228/318)	0.01	69.34%(224/323)	71.70%(228/318)	0.514
D5/D6	43.64%(2336/5353)	28.3%(90/318)	0.01	30.66%(99/318)	28.3%(90/318)	0.514
Implantation rate	42.4% (2267/5353)	38.7% (123/318)	0.198	40.6% (131/323)	38.7% (123/318)	0.627
biochemical pregnancy rate	57.5% (2115/3681)	57.2% (118/206)	0.960	56.3% (116/206)	57.2% (118/206)	0.842
Clinical pregnancy rate	53.8% (1979/3681)	53.4% (110/206)	0.919	52.4% (108/206)	53.4% (110/206)	0.844
Abortion rate	17.2% (340/1979)	18.2% (20/110)	0.787	19.4% (21/108)	18.2% (20/110)	0.811
Live birth rate	43.68% (1608/3681)	42.71% (88/206)	0.786	42.23% (87/206)	42.71% (88/206)	0.921
birth weight(g)	3327.65 ± 587.22	3381.70 ± 578.98	0.683	3341.55 ± 540.99	3381.70 ± 578.98	0.996
Multiple rate	15.85% (310/1955)	13.89% (15/108)	0.585	21.30% (23/108)	13.89% (15/108)	0.153

Patient characteristics, such as gravidity, and duration of infertility, FSH level and LH level were similar between the two groups. Age, BMI, AMH level were different before PSM but not after PSM. During controlled ovulation induction, the total dose and days of recombinant human FSH administration, numbers of retrieved oocytes, number of good-quality embryos, number of blastocysts, and number of embryos transferred between the two groups were also similar after PSM. After PSM, the number of oocytes retrieved in the control group was greater than that in the HSC-P patients (13.91 ± 8.62 *vs*. 10.99 ± 6.55, p< 0.001), which is more likely due to the higher proportion of OHSS in the control group. The pregnancy outcomes of groups 1 and 2 showed no significant difference in implantation rate, biochemical pregnancy rate, abortion rate, clinical pregnancy rate (53.8% *vs.* 53.4%, p=0.919 before PSM, 52.4% *vs.* 53.4%, p=0.844 after PSM), live birth rate (83.1% *vs.* 80%, p=0.743 before PSM, 80.6% *vs.* 80%, p=0.918 after PSM) or multiple pregnancy rate.

A subgroup analysis of the study group (**Table 2**) compared patients who received FET within different menstrual cycles. The three subgroups had similar baseline parameters. During FET cycles (**Table 3**), the biochemical pregnancy rates of the three subgroups were similar, as was the clinical pregnancy rate per cycle, spontaneous abortion rate and birth weight between the three subgroups. The live birth rate in patients with FET after more than 3 menstrual cycles was lower than that in patients with FET after less than 3 menstrual cycles, but the differences were not statistically significant. There was no significant difference in perinatal outcomes including very preterm delivery (VPTD), preterm delivery (PTD), low birth weight (LBW), macrosomia, small for gestational age (SGA), large for gestational age (LGA), birth weight(g), birth-height(cm)and Apgar Scores.

**Table 2:** Subgroup analysis of polypectomy group.

Parameter	FET after next menses(n = 137)	FET after 2 or 3 menstrual cycles(n = 40)	FET after >3 menstrual cycles(n = 29)	*p* value
Age (years)	33.00 [29.00, 38.00]	32.00 [30.00, 35.00]	30.00 [29.00, 35.00]	0.237
BMI (kg/m^2^)	23.70 [22.00, 26.70]	24.10 [22.00, 26.10]	22.90 [21.30, 25.70]	0.405
Gravidity	1.00 [0.00, 2.00]	1.00 [0.00, 2.00]	1.00 [0.00, 2.00]	0.739
**OPU cycle**				
Peak E2(nmol/l)	136445.48 ± 6843.75	145739.58 ± 7109.47	14116.33 ± 6903.57	0.527
Oocyte no. retrieved	10.632 ± 6.13	11.255 ± 6.68	12.347 ± 7.93	0.425
**Endometrial preparation**				0.872
Natural cycle	45.98% (63/137)	47.50% (19/40)	41.38% (12/29)	
Artificial cycle	54.01% (74/137)	52.5% (21/40)	58.62% (17/29)	
**Endometrial thickness**	10.43 ± 2.54	9.80 ± 2.04	9.75 ± 2.62	0.891
**ET no. Of embryoes**	2.000[1.000, 2.000]	2.000 [1.000, 2.000]	2.00 [1.000, 2.000]	0.624
**Development stage of the embryo**				0.989
D3	71.96% (154/214)	71.18% (42/59)	71.11% (32/45)	
D5	28.04% (60/214)	28.81% (17/59)	28.89% (13/45)	
**Good embryo no.**	2.00 [1.00, 2.00]	2.00 [1.00, 2.00]	2.00 [1.00, 2.00]	0.405

Baseline characteristics of patients undergoing FET cycles after hysteroscopic polypectomy, stratified by number of menstrual cycles.

**Table 3:** Outcomes analysis of Subgroup.

Parameters	FET after next menses (n = 137)	FET after 2 or 3 menstrual cycles(n = 40)	FET after >3 menstrual cycles(n = 29)	*p* value
Implantation rate	39.25% (84/214)	40.68% (24/59)	33.33%(15/45)	0.715
biochemical pregnancy rate	58.33% (80/137)	57.40% (23/40)	51.72% (15/29)	0.804
Clinical pregnancy rate	55.47% (76/137)	52.5% (21/40)	44.83% (13/29)	0.575
Early Abortion rate	18.42% (14/76)	14.29% (3/21)	23.08% (3/13)	0.808
Live birth rate	44.53% (61/137)	42.5% (17/40)	34.48% (10/29)	0.610
Newborn’s sex				0.701
Male	52.46% (32/61)	52.94% (9/17)	60% (6/10)	
Female	47.54% (29/61)	47.06% (8/17)	40% (4/10)	
very preterm delivery,<37weeks	1.64% (1/61)	0 (0/17)	0 (0/10)	0.543
Preterm delivery, <37weeks	4.92% (3/61)	5.88% (1/17)	0 (0/10)	0.613
Low birth weight	3.28% (2/61)	0 (0/17)	0 (0/10)	0.383
Small for gestational age	6.56%(6/61)	5.88% (1/17)	0 (0/10)	0.294
Large for gestational age	13.11% (8/61)	11.76% (2/17)	10%(1/10)	0.781
Macrosomia	11.48% (7/61)	11.76% (2/17)	10%(1/10)	0.942
twins	12.00% (9/75)	20% (4/20)	15.38% (2/13)	0.646
birth weight(g)	3383.33 ± 593.38	3402.78 ± 601.34	3200.00 ± 141.42	0.900
birth-height(cm)	50.15 ± 2.35	50.21 ± 2.09	49.91 ± 2.22	0.433
1-minute Apgar Scores	10 ± 0.3	10 ± 0.2	10 ± 0.2	0.743
10-minute Apgar Scores	10 ± 0.3	10 ± 0.2	10 ± 0.3	0.522

Pregnancy outcomes of patients undergoing FET cycles after hysteroscopic polypectomy, stratified by number of menstrual cycles.

## Discussion

Endometrial polyps are common benign endometrial lesions in gynaecology and are considered to be an important factor leading to female infertility (19). Endometrial polyps occur in 10% of asymptomatic women, 26% of women with unexplained low fertility, and 47% of women with endometrioid-related low fertility (3). HOXA10 and HOXA11 are downregulated in endometrial polyps, which may provide a molecular basis for reducing the pregnancy rate (20). Our centre does not require hysteroscopy before IVF, but we strongly recommend hysteroscopy for patients with a history of ch an incidence of 5.30%. The incidence was lower than expected, possibly because patients with endometrial abnormalities indicated by ultrasound had undergone HSC-P prior to IVF, and these patients were excluded from this research.

Endometrial polyps are mostly asymptomatic, so most endometrial polyps are diagnosed accidentally by routine gynaecological examination, such as *via* hysteroscopy or transvaginal ultrasound. Therefore, routine hysteroscopy is necessary for infertile patients before assisted reproduction treatment (21, 22). Contrary evidence has also been reported. Isikoglu ET et al.’s retrospective case–control study showed that polyps less than 1.5 cm in diameter had no significant adverse effect on IVF-ET/ICSI transplantation and pregnancy outcomes (23). Our data show that the pregnancy rate of FET after HSC-P is not different from that of patients without a diagnosis of endometrial polyps after hysteroscopy. The exact mechanism of endometrial polyp formation and infertility is not clear. The formation of endometrial polyps may be related to inflammation and mechanical damage, local hormone environment disturbance, excessive growth of the basal layer endometrium, cell proliferation and apoptosis imbalance (24). The above mechanisms are not unique to polyps but also play an important role in the pathogenesis of endometriosis and uterine fibroids. Perhaps these factors also affect the pregnancy rate after FET in patients with endometrial polyps. However, our current data support that HSC-P is necessary for patients with endometrial polyps.

In recent years, with the improvement of embryo freezing technology, FET has been widely used in IVF-ET. Multiple studies have shown that FET cycles are associated with a live birth rate similar to or even higher than that of fresh transplant cycles and significantly reduced risk of ovarian overstimulation, increased cumulative pregnancy rate, and reduced rate of ectopic pregnancy (25, 26). Although FET also has its disadvantages, such as a high incidence of hypertension during pregnancy and large gestational age (20, 27), an increasing number of fertility centres around the world have begun to use FET. Although current evidence suggests that endometrial polyp resection is beneficial before pursuing assisted reproduction, there are only a few studies regarding the optimal timing of FET. Research has demonstrated that when frozen-thawed blastocyst transfers are performed within an interval of 120 days after polypectomy, there are higher biochemical pregnancy rates and clinical pregnancy rates compared with intervals greater than 120 days (28). Notably, this study was performed on frozen-thawed blastocyst transfers only, which may not be directly applicable to fresh cycles. Given the purported evidence that endometrial polyps can develop in conditions associated with increased or unopposed E_2_ levels, as in the case of ovarian stimulation during IVF, estrogenic stimulation of the endometrium plays a significant role in their development (29). Therefore, we selected patients who underwent HSC-P after whole embryo freezing to eliminate the effects of COS.

First, our results suggest that HSC-P is necessary. Previous studies have confirmed higher implantation and pregnancy rates after mild endometrial injury in the menstrual cycle preceding IVF (30, 31). However, the conclusion of current studies is not clear about the repair time of endometrial injury after HSC-P. There are studies that suggest that the success rates may not be superior if the treatment is started in the first few months postoperatively in IVF (14). The controversy about endometrial damage and repair time after hysteroscopy has not been settled, especially for FET. Whereas our study stratified the time period between polypectomy and FET cycle start by the number of intervening menstrual cycles, the results of subgroup analysis suggested that compared with FET after their next menses, waiting for two or more menstrual cycles after HSC-P does not necessarily produce superior outcomes. This may be due to a combination of corrected uterine pathology and the potentially beneficial effects of endometrial injury caused by HSC-P, similar to endometrial scratching (32, 33). Due to the limited number of patients, patients with more than 3 menstrual cycles were not eligible for subgroup analysis, so they were classified into the third subgroup. Seven of the patients were transplanted during the fourth or fifth menstrual cycle, and it must be noted that the reasons for delaying FET were largely logistical or personal. We do not know whether these factors continue to influence subsequent transplants. This may explain the lower live birth rate in the third subgroup.

The strengths of the present study include its sample size, which is larger than previously published studies. In addition, we included patients who underwent hysteroscopy after controlled ovulation stimulation, which eliminated the influence of ovulatory drugs and ovulatory time on the outcome and more accurately reflected the influence of HSC-P on the pregnancy rate of FET. Compared to previous studies, we increased neonatal outcomes. However, our data only suggest that there is no need to delay FET due to HSC-P, and we were unable to explore whether postponing transplantation would affect the success rate of FET.

## Conclusion

The data from this study suggest that the time between HSC-P and the start of FET cycles does not affect cycle outcomes. Waiting for two or more menstrual cycles after surgery did not produce superior outcomes.

## Data availability statement

The raw data supporting the conclusions of this article will be made available by the authors, without undue reservation.

## Author contributions

BW and NM designed the study and wrote the manuscript. CR and YG designed the study, and PK and WZ analyzed the data. All authors contributed to the article and approved the submitted version.

## Funding

This work was supported by grant from Joint Project of Medical Disciplines of Henan Province [LHGJ20200428, BW; LHGJ20200435, WZ; LHGJ20220538, NM] and PhD research startup foundation of the Third Affiliated Hospital of Zhengzhou University [grant number: 2021082].

## Conflict of interest

The authors declare that the research was conducted in the absence of any commercial or financial relationships that could be construed as a potential conflict of interest.

## Publisher’s note

All claims expressed in this article are solely those of the authors and do not necessarily represent those of their affiliated organizations, or those of the publisher, the editors and the reviewers. Any product that may be evaluated in this article, or claim that may be made by its manufacturer, is not guaranteed or endorsed by the publisher.
